# Situation of Rabies in Ethiopia: A Five-Year Retrospective Study of Human Rabies in Addis Ababa and the Surrounding Regions

**DOI:** 10.1155/2021/6662073

**Published:** 2021-02-19

**Authors:** Mesfin Aklilu, Wogayehu Tadele, Amelework Alemu, Sintayehu Abdela, Garuma Getahun, Alemnesh Hailemariam, Yirgalem Tadesse, Gutu Kitila, Endalkachew Birhanu, Ibsa Fli, Abebe Getachew, Yimer Mulugeta

**Affiliations:** Ethiopian Public Health Institute (EPHI), Addis Ababa, Ethiopia

## Abstract

**Objective:**

The study objective was to estimate the burden of human rabies in Ethiopia from 2015–2019. *Study Design*. A descriptive study design was applied to measure the size of the problem.

**Method:**

Retrospective data were used from the Ethiopian Public Health Institute rabies case record book that was registered between 2015 to 2019.

**Result:**

Eighty-seven (87) cases of human rabies were diagnosed clinically in the Ethiopian Public Health Institute over the period of five years (2015–2019) with 100% case fatality. Of these, 83 (95.4%) cases were attributed to dog bites, whereas 1 (1.1%) to a cat and 3 (3.4%) to wild animals. The fatalities were from Oromia (*n* = 51 (58.6%), 13 (14.9%) were from Amhara, 15 (17.2%) were from Addis Ababa, and 8 (9.2%) from the Southern region. All referred cases had no record of immunization against rabies except eight. Ineffective postexposure treatment was the reason for 5 (5.7%) deaths. Out of 1,652 brain samples of different animals, mainly dogs, submitted for examination, 1,122 (68%) were found to be positive for rabies by the FAT. Dog bites were more common among males than females. The number of dog bite victims who had visited the EPHI counseling office and recommended to take postexposure prophylaxis against rabies both from Addis Ababa and the surrounding areas were 9,592 and 4,192, respectively. Out of these, 5,708 were males and 3,884 females for the capital Addis Ababa. Similarly, 2,439 males and 1,753 females account for areas surrounding Addis Ababa. Among those exposed from Addis Ababa, 1,079 (11.2%) were in the age group less than five, 1696 (17.7%) were in the age group 6–13, and 6,817 (71.1%) in the age group 14 and greater. Victims from outside of the capital Addis Ababa account for 644 (15.4%) for the age group less than 5 years, 964 (23%) for the age group 6–13 and, 2,584 (61.6%) for the age group 14 and greater.

**Conclusion:**

Mechanisms must be sought to reduce the cost of PEP and means of obtaining funds so as to initiate timely treatment for rabies exposed individuals of low socioeconomic status. Besides prevention, strategies should focus on public education and strict dog population control.

## 1. Introduction

Ethiopia is a big country with a population of 99.39 million and is composed of nine regional states and two chartered city administrations. The average income per capita is 13.92 USD [1]. Rabies is one of the most feared infectious diseases worldwide, predominantly occurring in Asia and Africa, where rabies is endemic in domestic dog populations [2]. Rabies causes at least 24,000 deaths per year in Africa [3]. The study done in Ghana, in Techiman municipality, recorded 13 cases of human rabies diagnosed clinically with 100% case fatality over a period of six years (2011 to 2016) [4]. The most fatal of all infectious diseases remains a major public health problem and causes severe economic impact in many developing countries like Ethiopia, regardless of the availability of effective vaccines for its treatment [5]. The fatal human cases during the period of study were 386 humans with an annual range of 35 to 58 persons per year. The overall number of postexposure treatments for humans was 17,204 within and around Addis Ababa. During the same period, 20,414 suspected rabid animals were clinically examined; nevertheless, only 10% were positive for rabies. Among 3,460 animal brains investigated in the laboratory with FAT, 75% were confirmed as rabies positive. The production and distribution of nerve tissue antirabies vaccine produced locally by the Ethiopian public health institute was 130,673 treatment doses for human vaccine and 85,055 doses for animal vaccine, respectively, from 2001 to 2009 [6]. Annual estimated rabies incidence of 2.33 cases per 100,000 in humans, 412.83 cases per 100,000 in dogs, 19.89 cases per 100,000 in cattle, 67.68 cases per 100,000 in equines, and 14.45 cases per 100,000 in goats were recorded [7]. Evidence suggests that 99% of human deaths were caused by dog bites [3]. Dog bites are a serious public health problem that inflicts considerable physical and emotional damage on victims and incurs immeasurable hidden costs to communities [8]. In sub-Saharan Africa, most of the rabies cases in animals and humans are caused by the canine rabies virus [9]. Although rabies is recorded in 150 territories and is responsible for at least 60,000 human deaths every year worldwide, it remains a neglected tropical problem [10]. Retrospective information recorded at EPHI during the period 1990–2000 indicates that canine rabies is a well-established disease in Addis Ababa, with no decline in the annual number of confirmed rabid cases. The dog population in Addis Ababa is estimated to be around 230,000–300,000, out of which 30% are owned while 70% are ownerless (stray dogs) [11]. According to the Ethiopian Public Health Institute/EPHI/report (1990–2000 E.C.) about 22,219 people in Addis Ababa city and its surrounding received postexposure treatment following dog bites (about 2200 people annually). In developing countries where financial resources are limited and there are numerous competing interests, there is a need for quantitative data on the public health burden and costs of diseases to support intervention prioritization [12]. There is a need to implement dog bites surveillance system and strengthen rabies surveillance in the context of the existing integrated disease surveillance and response system (IDSR) to improve early response to the disease [4]. Rabies vaccines produced in mammalian neural tissues (NTV) have the disadvantage of causing severe adverse reactions, at a rate estimated as 0.3 0.8 per thousand treated patients [13]. WHO issued a resolution for the complete replacement of nerve tissue vaccines by 2006 with cell culture rabies vaccines. However, sheep brain derived Fermi type rabies vaccine is still being manufactured and utilized for the majority of exposed patients in Ethiopia [13]. The 2018 update of the WHO position on rabies immunization is an important step towards improving public health outcomes for rabies, increasing health equity and ultimately reaching the global goal of zero human rabies deaths by 2030 worldwide [14]. A number of factors have come together to make this an opportunity time to undertake rabies control and elimination strategy. These factors include the establishment of a One Health Office in the country and increased interest in rabies elimination by partners. A pilot project was designed by the joint efforts of the University of Gondar, Ethiopian Public Health Institute, Ohio State University, and the US Centers for Disease Control and Prevention, with the aim to control canine rabies in a northern Gonder of Ethiopia which can be used for further scale-up. The other opportunity is the ability for local production of safe and effective modern rabies vaccines for both animals and humans. Modern cell culture antirabies vaccine production for animal use has been transferred to National Veterinary Institute from the Ethiopian Public Health Institute for mass production. For human purposes, the effort to replace the Fermi type with modern cell culture vaccine is in progress at EPHI and currently, preclinical trials were finalized. In the coming two years, it is assumed to replace the Fermi vaccine, which is expected to contribute to the control and elimination of the disease in Ethiopia [9].

## 2. Materials and Methods

Retrospective written records of all the patients who visited the institute: those individuals exposed to animals suspected of rabies and history of the exposing animals from January 2015 to October 2019 were reviewed for the study. Four case recording books were reviewed.

- Human fatal rabies case recording log books: used to assess the demographic features of the patients, clinical signs and symptoms, lengths of illness, date of bite, site of the bite, history of postexposure prophylaxis, vaccinal complication, incubation period, and date of death
- Postexposure prophylaxis treatment log books for Addis Ababa and outside of Addis Ababa city: It consists of information about demographic features of the bitten person, area of residence, site of the bite, date of the bite, category of exposure, ownership status of the animal, first aid treatment taken, PEP prescribed, and health facility referred.
- Animal rabies diagnosis registration log books: Name and full addresses of the owners of the responsible animal, vaccination status of the animal, full address of the victims, site of the bite, and any treatment administered to the victims clinical diagnosis result.
- Quarantined animals and first aid treatment registration log books: This consists of data about the ownership status of the animal that allows for a decision either to control or observe the animal for ten days regarding owned dogs (Quarantine at home) or to euthanize the animal and confirm the disease in the laboratory as far if the dog is unowned. It also consists of 10 days quarantine result. It also consists of data pertaining to wound care management, full address of both the owner of the animal and the victim to ensure proper follow-up.

Data obtained from the aforementioned documents were entered into Epi Info version 7 and transferred to SPSS version 20 for analysis. Descriptive statistics were used to analyze the data.

## 3. Result

### 3.1. Rabies Fatality

From a total of 87 fatal rabies cases recorded in The Ethiopian Public Health Institute, human and animal rabies diagnosis and consultation office between January 2015 and October 2019, 83 (95.4%) were attributed to dogs whereas 1 (1.1%) to cat, and 3 (3.4%) to wild animals. History of exposure was partially available for all patients. All referred cases had no record of immunization against rabies except eight cases. Ineffective postexposure treatment was the reason for 5 (5.7%) deaths. Out of the total deaths, 40 (46%) were males and 47 (54%) females. Their median age was 22 years; 6 (6.9%) were under 5 years of age, 18 (20.7%) between 11–20 years, and 45 (51.7%) of the deaths were greater than 20 years of age. Almost all patients manifested the typical clinical signs of rabies at presentation to the EPHI rabies counseling office. Aerophobia and hydrophobia were the typical symptoms that served as a parameter for diagnosing the disease. Anxiety and paralysis were also frequently observed. Almost all patients who visited the rabies counseling office had a history of dog bite except one case that was attributed to a cat bite/scratch, and three cases for hyena and fox prior to the onset of illness. 51 (58.6%) were from the Oromia region, 13 (14.9%) were from the Amhara region, 15 (17.2%) were from Addis Ababa, and 8 (9.2%) from the Southern region. Regarding exposure site, 27 (31%) were bitten on the legs, while 36 (41.4%), 13 (14.9%), and 10 (11.5%) on their hand, face, and other parts of the body. Reported injuries were both by stray and owned dogs, a cat, and wild animals. None of them had any history of prior antirabies vaccination. Thirty-six (41.4%) of the animals that people were exposed to were unknown, whereas 6 (6.9%) were owned but disappeared after infecting the patient. Twelve (13.8%) of the exposing animals died after paralysis, whereas 33 (37.9%) were killed immediately after bite contact. The patients' history revealed that 6 (6.9%) and 2 (2.3%) individuals were started PEP (NTV) and cell culture vaccines and discontinued prior to completing the course. Among the victims, 2 (25.3%) used traditional treatments, and 57 (65.5%) had no treatment against rabies. 5 (5.7%) of the cases had shown vaccinal complications due to the side effects that arose from the use of nerve tissue vaccine (NTV), whereas 82 (76.3) of them did not. As a result of the poor prognosis, all were advised to go back home with their families after learning the ineffective prognosis of the disease. Among patients clinically diagnosed with rabies, only two were admitted to the isolation ward of St. Paul Hospital for palliative care. Length of illness varied from 1 to 8 days. The source of history or referral was family members or friends. Patients were observed seeking care on their own initiative. The fatality rate recorded was 100% (Table 1).

### 3.2. Rabies Exposure

Dog bites were more common among males than females. Male individuals are more likely to be diagnosed with these disorders, engage in externalizing behaviors to a greater degree, and their behavior disorder symptoms tend to be more severe than female individuals. The number of dog bite victims who had visited the EPHI counseling office and recommended to take postexposure prophylaxis against rabies both from Addis Ababa and the surrounding areas were 9,592 and 4,192, respectively. Out of these, 5,708 (%) were males and 3,884 (%) females for the capital Addis Ababa. Similarly, 2,439 males and 1,753 females account for areas surrounding Addis Ababa (Table 2).

Among those exposed from Addis Ababa, 1,079 (11.2%) were in the age group less than five, 1696 (17.7%) were in the age group 6–13, and 6,817 (71.1%) in the age group 14 and greater. Victims from outside of the capital Addis Ababa account for 644 (15.4%) for the age group less than 5 years, 964 (23%) for the age group 6–13 and, 2,584 (61.6%) for the age group 14 and greater (Table 3).

### 3.3. Post Exposure Prophylaxis

**Figure 1:**
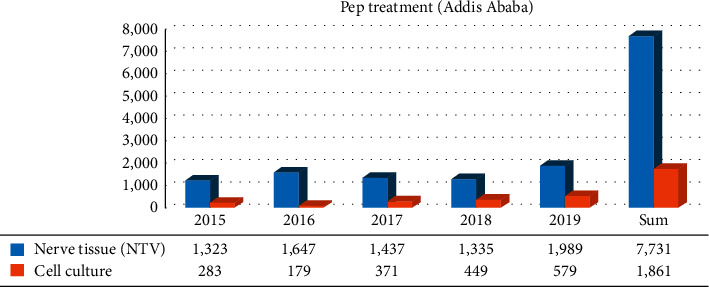
Postexposure prophylaxis treatment (PEP) against rabies between 2015 and 2019 in Addis Ababa.

**Figure 2:**
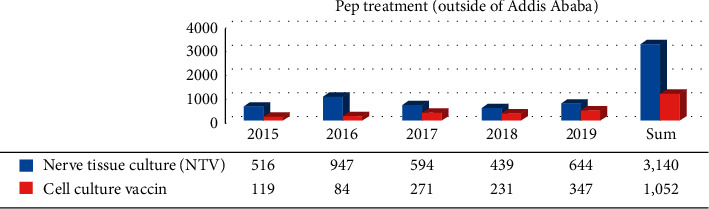
Postexposure prophylaxis treatment (PEP) against rabies between 2015 and 2019 outside of Addis Ababa.

A locally produced 5% suspension of phenolized sheep brain tissue vaccine (NTV) was prescribed as postexposure prophylaxis for 7,731 (80.6%) and 3,140 (74.9) victims from Addis Ababa and outside of Addis Ababa, respectively. The availability of the vaccine is not consistent. The current average cost of the vaccine in Ethiopia is 42.5 USD per vial. The total cost incurred for full dose vaccination would be 170 USD. Poor supply of the vaccine coupled with a high cost of the vaccine continues to pose an additional threat in Ethiopia. Cell culture antirabies vaccine was used for 1,861 (19.4%) and 1,052 (25.1%) dog bite victims from Addis Ababa and outside of Addis Ababa, respectively (Figures 1 and 2).

**Figure 3:**
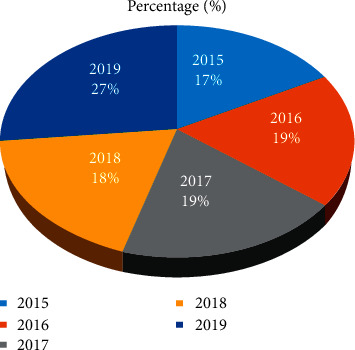
Percentage comparison of PEP treatment in Addis Ababa (2015–2019).

**Figure 4:**
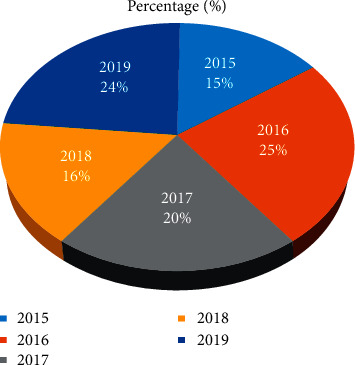
Percentage comparison of PEP treatment outside of Addis Ababa (2015–2019).

Percentage comparison of PEP treatment in the Addis Ababa region during the five-year period showed no significant difference except for the year 2019 (Figure 3). Unlike Addis Ababa, PEP treatment outside of Addis Ababa slightly differs during the periods (Figure 4).

## 4. Discussion

The study recorded 87 cases of human rabies diagnosed clinically in the Ethiopian Public Health Institute over the period of five years (2015–2019) with 100% case fatality (Table 1). However, there are two regional laboratories recently established in the Amhara and Tigray regional states by the joint planning and financial aid of the center for disease control (CDC). Due to certain logistical issues, the laboratories are not currently functioning in their full capacity. There is only one human rabies counseling office with a referral laboratory in the institute for rabies diagnosis service, which is based on animal clinical observation under ten days quarantine period and laboratory confirmation. The facility has a postmortem room and incinerator for carcass disposal. Fluorescent antibody technique (FAT) is applied for laboratory confirmation. The majority of human rabies cases in the present study came from rural communities of the Oromia region (58.6%) compared to the capital Addis Ababa (17.2%), Amhara region (14.9), and SNNP (9.2%). The majority involved male victims. This is in line with other African studies where human deaths often occur among people in rural farming communities with limited access to health-care resources [4]. Many studies showed that children are more likely to get bitten on the area of the head and neck [4]. Nonetheless, in the Ethiopian scenario, infants and school children who play closely with pets at home and even on the streets are more likely to be exposed than adults; the present study unlikely indicated that the largest proportion of age group was 20 and greater as far as human rabies case is concerned. The reaction of victims varies according to different age groups. In some cases, younger age groups do not care for the further preventive measure after they have received first aid treatment. To the contrary, some elders are well aware of the dangers of rabies and look for proper medical care [6]. Evidence suggests that the statistics in this research may, in fact, reflect a gross underreporting of the real magnitude of human rabies in the study area. This is explained by a misdiagnosis of the disease in the health-care facility, spiritual and traditional treatment seeking behavior that discourages victims from acquiring appropriate consultation and antirabies vaccination. Factors that contributed most to human rabies in Ethiopia include the use of traditional medicinal plants, spiritual beliefs, and poor treatment seeking behavior due to various misperceptions, and considering first aid as antirabies treatment as a remedy for rabies. The efficacy and protection of these commonly used antirabies folk drugs were not well demonstrated and understood in the country [5]. In addition, folk medicines (traditional herbal medicine) were choices for such a shame treatment of rabies in different parts of the country in areas where rabies vaccines are physically inaccessible and economically expensive [5]. Because of both geographical and cultural variations, the reliability of the incidence data reported is expected to vary among regions in Ethiopia. For example, in rural Ethiopia, people exposed to rabies frequently prefer to see traditional healers for the diagnosis and treatment of the disease than urban residents because of cultural background, lack of knowledge, or limited accessibility to medical treatment [12]. Thousands of human deaths from rabies occur every year after exposure, despite the accessibility of effective vaccines and the control of diseases in the animal reservoir. The cost of postexposure prophylaxis (PEP) and the regimen conveyed varied based upon the health-care facility and the date of delivery [15]. Similar studies previously conducted at the Ethiopian Institute of Public Health revealed that most fatal cases of human rabies recorded were associated with the use of herbal remedies whereby traditional healers exhaustively handled the majority of cases of human rabies. Meanwhile, most human rabies cases manifested before reaching health-care facilities [6]. The present study is consistent with the analysis carried out in Tanzania, where high PEP costs for patients without RIG are a major barrier that limits access [16]. Though WHO recommended the usage of tissue culture vaccine, due to economic reasons [14], nerve tissue vaccine (NTV) is widely used as postexposure prophylaxis in Ethiopia. Annually, 30,000 to 33,000 doses of NTV are produced by the Ethiopian public health institute. According to the previous vaccination trend, the total doses produced annually are assumed to satisfy the demand of the country. The cost of vaccination per individual ranged from 5 to 23 USD. No PEP surveillance system to monitor adverse events that arise from NTV and completion of a full course of treatment. Access to cell culture based vaccines in Ethiopia is restricted due to their high price (80 USD per individual). Rabies immunoglobulin (RIG) is not accessible within the country [17]. Low compliance with the NTV vaccination schedule results in receiving an incomplete course [18], misdiagnosis, inadequate knowledge of the mode of transmission of the diseases (for medical and veterinary professionals and for the general public), poverty, and the lack of proper affordable medical care, all resulting in needless human deaths [15]. The lack of institutional involvement contributes significantly to the persistent prevalence of human rabies in the country [19]. Mechanisms must be sought to lessen the cost of PEP and means of obtaining funds so as to early initiate treatment for rabies exposed individuals of low socioeconomic status. Evaluation of the use of economical intradermal PEP regimens for multiple patients could be an example [15]. Besides, prevention strategies should focus on public education and strict dog population control.

## Figures and Tables

**Table 1:** Classification of fatal human rabies cases according to different variables from Addis Ababa and the surrounding areas between 2015 and 2019.

Age	<5	5–10	11–20	>20	
Region	6 (6.9%)	18 (20.7%)	18 (20.7%)	45 (51.7%)	
	Oromia	Amhara	Addis Ababa	SNNP	Total *N*/%
	51 (58.6%)	13 (14.9%)	15 (17.2%)	8 (9.2%)	87 (100%)
Site of exposure	Leg	Hand	Face	Other	*N*/%
	27 (31%)	36 (41.4%)	14 (16.1%)	10 (11.5%)	87 (100%)
Exposing animal	Dog	Cat	Other		*N*/%
	83 (95.4%)	1 (1.1%)	3 (3.4%)		87 (100%)
History of exposing animal	Unknown	Killed	Died	Disappeared	*N*/%
	36 (41.4%)	33 (37.9%)	12 (13.8%)	6 (6.9%)	87 (100%)
Treatment taken	NTV	Tissue culture	Traditional	No treatment	*N*/%
	6 (6.9%)	2 (2.3%)	22 (25.3%)	57 (65.5%)	87 (100%)
Length of illness	Minimum	Maximum	Mean	St. deviation	*N*/%
	1	8	3.43	0.16	87 (100%)
Patient status	Died	Survived	N/%		
	87	—	87 (100%)		
Vaccinal complication	Yes	No	N/%		
	5	82	87 (100%)		

**Table 2:** Sexwise distribution of PEP treatment against rabies between 2015 and 2019 in Addis Ababa and outside Addis Ababa.

Year	Sex			
	Addis Ababa		O/Addis Ababa	
	M	F	M	F
2015	934	672	384	251
2016	1,044	782	593	438
2017	1,072	736	512	353
2018	1,081	703	362	308
2019	1,577	991	588	403
Sum/%	5,708 (59.5%)	3,884 (40.5%)	2,439 (58.2%)	1,753 (41.8%)

**Table 3:** Age wise distribution.

Year	Age group					
	Addis Ababa			Out of Addis Ababa		
	≤5	6–13	14 and above	≤5	6–13	14 and above
2015	147	292	1,167	101	153	381
2016	198	353	1,275	137	264	630
2017	283	408	1,117	150	174	541
2018	130	266	1,388	101	162	407
2019	321	377	1,870	155	211	625
Sum/%	11.2 (1,079)	17.7% (1,696)	71.1% (6,817)	15.4% (644)	23% (964)	61.6% (2,584)

## Data Availability

The data that form the basis of this paper are available upon reasonable request to the corresponding author.
